# Screening intimate partner violence in the healthcare services during Covid-19 lockdowns in Israel: Beatris Pekar Agronsky

**DOI:** 10.1093/eurpub/ckac130.008

**Published:** 2022-10-25

**Authors:** B Pekar Agronsky, N Daoud

**Affiliations:** Public Health, Ben-Gurion University of the Negev, Beer-Sheva, Israel

## Abstract

**Background:**

Studies have shown increased rates of intimate partner violence (IPV) during the Covid-19 lockdowns. Healthcare services (HCS) have an important role in detection and screening of women victims of IPV. These women tend to visit the HCS more frequently, which creates an opportunity to detect, screen, and inform them about relevant support services.

**Methods:**

We conducted an online survey during Israel's 2nd and 3rd lockdowns (October 2020-February 2021). A self-administrated structured questionnaire was distributed in Arabic and Hebrew via social media. Eligibility criteria included women >18 years old. 519 women completed the questionnaire: Palestinian-Arab=73; non-immigrant Jew=319; and immigrant Jew=127. We asked women whether they were ever screened (ES) for IPV or received information (RI) on support services in the HCS.

**Results:**

37.2% of the women reported any IPV; Palestinian-Arab women reported higher rates of IPV (49.3%) compared to non-immigrant Jew (34.2%) and immigrant Jew (37.8%). Prevalence of ES and RI on support services were low among the total study sample (21.8%, 47% respectively). Only 12.1% reported on both (ES and RI). Among women who reported IPV, only 26.9% reported that they had been ES, 39.4% RI, and 13.5% both. Whilst Palestinian-Arab women victims of IPV reported higher ES and a lower RI (30.6%,25% respectively) non-immigrant and immigrant Jew reported the opposite -higher prevalence of RI and less ES (non-immigrant Jew 45%,25.7%. Immigrant Jew 37.5%,27%, respectively). In the multivariate analysis after adjusts, Palestinian-Arab women were less likely to RI regarding support services (OR = 0.33,90%CI=0.19-0.57), while immigrant Jew women had a greater chance to be ES in HCS (OR = 4.29, 90% CI=1.43-12.80).

**Conclusions:**

To increase IPV detection in the HCS, there is a need for interventions on screening and providing information on support services specifically during emergencies where IPV is likely to increase.

**Key messages:**

- During emergency situations such as the Covid-19 when IPV is likely to increase, HCS should make more efforts for IPV screening and providing information on support services to women victims of IPV.
- Tailored intervention should consider barriers in the HCS for providing less information on support services to minority women, and less screening for immigrant women.

